# Supplementary material for: Gene synthesis allows biologists to source genes from farther away in the tree of life
Source: Nat Commun. 2018 Oct 24;9:4425. doi: 10.1038/s41467-018-06798-7 (PMC6200774; doi:10.1038/s41467-018-06798-7)
Supplement: Supplementary file 1 — Supplementary Information [file 41467_2018_6798_MOESM1_ESM.pdf]

Supplementary Information

"Gene synthesis allows biologists to source genes from farther away in the tree of life"

Aditya M. Kunjapur[1,2†*], Philipp Pfingstag[3,4†], Neil C. Thompson[5,6*]

1. Department of Chemical Engineering, Massachusetts Institute of Technology, Cambridge, MA, 02142 USA

2. Present Address: Department of Chemical and Biomolecular Engineering, University of Delaware, Newark, DE, 19716, USA

3. Sloan School of Management, Massachusetts Institute of Technology, Cambridge, MA, 02142, USA

4. Present Address: School of Management, Technical University of Munich, D-80333, Munich, Germany

5. Computer Science and Artificial Intelligence Lab (CSAIL), Massachusetts Institute of Technology, Cambridge, MA, 02142, USA

6. Laboratory for Innovation Science at Harvard (LISH), Harvard University, Cambridge, MA, 02138, USA

† Contributed equally

* Corresponding authors: AMK (kunjapur@udel.edu) or NT (neil_t@mit.edu)

Consists of:

Supplementary Figures

Supplementary Tables

Supplementary Methods

Supplementary Discussion

Supplementary References

Supplementary Figures

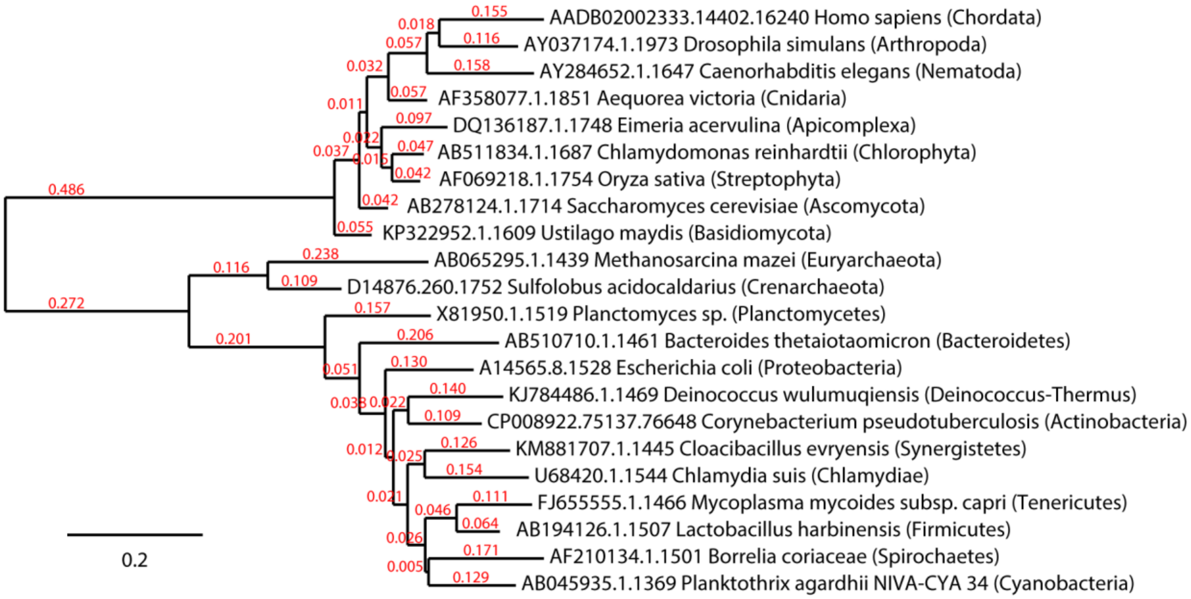

Supplementary Figure 1. Representative organisms for each phylum and associated accession number of 16S/18S rRNA sequence. Phylogenetic tree was generated using the online tool Phylogeny.fr. Red numbers indicate branch lengths, which are related to nucleotide substitutions per site and were used for genetic distance estimation.

Supplementary Tables

Supplementary Table 1. Codon-substitution calculation. Note that natural amino acid frequencies come from literature[1].

**Amino Acid**	**Codon Choices**	**% Sequence Identity**	**Natural Frequency (%)**	**Weighted %Id**
A	4	75%	7.8	6%
R	6	61%	5.23	3%
N	2	83%	4.37	4%
D	2	83%	5.19	4%
C	2	83%	1.1	1%
Q	2	83%	3.45	3%
E	2	83%	6.72	6%
G	4	75%	6.77	5%
H	2	83%	2.03	2%
I	3	78%	6.95	5%
M	1	100%	2.28	2%
L	6	61%	10.15	6%
K	2	83%	6.32	5%
F	2	83%	4.39	4%
P	4	75%	4.26	3%
S	6	46%	6.46	3%
T	4	75%	5.12	4%
W	1	100%	1.09	1%
Y	2	83%	3.3	3%
V	4	75%	7.01	5%
**Average**	3.1	78%	**Weighted Average**	75.1%

Supplementary Table 2. Percentage identity expected after codon-substitution for arginine

Arginine (R)	Codon after substitution						
Starting Codon	CGT	CGC	CGA	CGG	AGA	AGG	Average
CGT	100%	67%	67%	67%	33%	33%	61%
CGC	67%	100%	67%	67%	33%	33%	61%
CGA	67%	67%	100%	67%	67%	33%	67%
CGG	67%	67%	67%	100%	33%	67%	67%
AGA	33%	33%	67%	33%	100%	67%	56%
AGG	33%	33%	33%	67%	67%	100%	56%
						**FINAL**	61%

Supplementary Table 3. Percentage identity expected after codon-substitution for serine

Serine (S)	Codon after substitution						
Starting Codon	TCT	TCC	TCA	TCG	AGT	AGC	Average
TCT	100%	67%	67%	67%	33%	0%	56%
TCC	67%	100%	67%	67%	0%	33%	56%
TCA	67%	67%	100%	67%	0%	0%	50%
TCG	67%	67%	67%	100%	0%	0%	50%
AGT	33%	0%	0%	0%	100%	67%	33%
AGC	0%	33%	0%	0%	67%	100%	33%
						**FINAL**	46%

Supplementary Table 4. *E. coli* training set sequences.

Organism for Expression	Addgene Sequence	ORF Frame	Natural or Synthetic
E. coli	6762	ORF frame 1 (1528-3411)	Synthetic
E. coli	67070	ORF frame 2 (1610-2794)	Natural
E. coli*	67070	ORF frame 2 (2822-3988)	Natural
E. coli*	67070	ORF frame 3 (4014-5297)	Natural
E. coli	67070	ORF frame 2 (5729-7060)	Synthetic
E. coli	67070	ORF frame 1 (7081-8436)	Synthetic
E. coli	67070	ORF frame 3 (8457-9647)	Natural
E. coli	67070	ORF frame 2 (9662-10216)	Natural
E. coli	67070	ORF frame 3 (10515-11408)	Synthetic
E. coli	67070	ORF frame 3 (11433-13064)	Synthetic
E. coli	68901	ORF frame 3 (1437-2192)	Synthetic
E. coli	74623	ORF frame 1 (1039-2202)	Natural
E. coli	74623	ORF frame 3 (2385-3380)	Natural
E. coli	74623	ORF frame 3 (3564-6227)	Natural
E. coli	74623	ORF frame 2 (6413-8305)	Natural
E. coli	74623	ORF frame 1 (8488-9912)	Natural
E. coli	109895	ORF frame 1	Natural
E. coli	114599	ORF frame 3 (72-1031)	Natural
E. coli	114599	ORF frame 2 (1214-1684)	Natural
E. coli	114599	ORF frame 1 (1867-3216)	Natural
E. coli	114599	ORF frame 3 (3399-4313)	Natural
E. coli	118300	Full sequence, reverse complement	Synthetic
E. coli	127060	ORF frame 1	Natural
E. coli	142299	ORF frame 2 (1255-1764)	Natural
E. coli	142299	ORF frame 1 (191-1258)	Natural
E. coli	151486	ORF frame 1 (4147-7278)	Synthetic
E. coli	152940	ORF frame 1 (2686-3339)	Natural
E. coli	152940	ORF frame 3 (3603-4703)	Natural
E. coli	6762	ORF frame 1 (1528-3411)	Synthetic
E. coli	67070	ORF frame 2 (1610-2794)	Natural
E. coli*	67070	ORF frame 2 (2822-3988)	Natural
E. coli*	67070	ORF frame 3 (4014-5297)	Natural
E. coli	67070	ORF frame 2 (5729-7060)	Synthetic
E. coli	67070	ORF frame 1 (7081-8436)	Synthetic
E. coli	67070	ORF frame 3 (8457-9647)	Natural
E. coli	67070	ORF frame 2 (9662-10216)	Natural
E. coli	67070	ORF frame 3 (10515-11408)	Synthetic

*In two of these cases, which are marked by asterisks, sequences that were supposedly codon-optimized appeared instead to be amplified from genomic DNA, as revealed through the papers they cited in their methods section and by the 100% sequence identity with natural sequences observed during BLAST.

Supplementary Table 5 *S. cerevisiae* training set sequences.

Organism for Expression	Addgene Sequence	ORF Frame	Natural or Synthetic
S. cerevisiae	146502	ORF frame 1	Synthetic
S. cerevisiae	108806	ORF frame 3	Synthetic
S. cerevisiae	97872	ORF frame 3	Synthetic
S. cerevisiae	56591	ORF frame 3	Natural

Supplementary Table 6. *S. cerevisiae* training set sequences.

Organism for Expression	Internal ID	Link	Natural or Synthetic
S. cerevisiae	Training_001	Randomly obtained on http://www.yeastgenome.org/browse/	Natural
S. cerevisiae	Training_002		Natural
S. cerevisiae	Training_003		Natural
S. cerevisiae	Training_004		Natural
S. cerevisiae	Training_005		Natural
S. cerevisiae	Training_006		Natural
S. cerevisiae	Training_007		Natural
S. cerevisiae	Training_008		Natural
S. cerevisiae	Training_009		Natural
S. cerevisiae	Training_010		Natural
S. cerevisiae	Training_011		Natural
S. cerevisiae	Training_012		Natural
S. cerevisiae	Training_013		Natural
S. cerevisiae	Training_014		Natural
S. cerevisiae	Training_015		Natural
S. cerevisiae	Training_016		Natural
S. cerevisiae	Training_017		Natural
S. cerevisiae	Training_018		Natural
S. cerevisiae	Training_019		Natural
S. cerevisiae	Training_020	Randomly obtained on http://www.yeastgenome.org/browse/ and codon-optimized for S. cerevisiae on https://eu.idtdna.com/CodonOpt	Synthetic
S. cerevisiae	Training_021		Synthetic
S. cerevisiae	Training_022		Synthetic

S. cerevisiae	Training_023		Synthetic
S. cerevisiae	Training_024		Synthetic
S. cerevisiae	Training_025		Synthetic
S. cerevisiae	Training_026		Synthetic
S. cerevisiae	Training_027		Synthetic
S. cerevisiae	Training_028		Synthetic
S. cerevisiae	Training_029		Synthetic
S. cerevisiae	Training_030		Synthetic
S. cerevisiae	Training_031		Synthetic
S. cerevisiae	Training_032		Synthetic
S. cerevisiae	Training_033		Synthetic
S. cerevisiae	Training_034		Synthetic
S. cerevisiae	Training_035		Synthetic
S. cerevisiae	Training_036		Synthetic
S. cerevisiae	Training_037		Synthetic
S. cerevisiae	Training_038		Synthetic
S. cerevisiae	Training_039		Synthetic

Supplementary Table 7. *H. sapiens* training set sequences.

Organism for Expression	Addgene Sequence or link	ORF Frame	Natural or Synthetic
H. sapiens	http://genomics.senescence.info/genes/allgenes.php - AF000237		Natural
H. sapiens	http://genomics.senescence.info/genes/allgenes.php - M91464		Natural
H. sapiens	72211	ORF frame 1	Synthetic
H. sapiens	124532	ORF frame 1	Synthetic
H. sapiens	117892	ORF frame 2	Synthetic
H. sapiens	125330	ORF frame 3	Synthetic
H. sapiens	142415	ORF frame 3	Synthetic
H. sapiens	78432	ORF frame 3	Synthetic
H. sapiens	137791	ORF frame 2	Synthetic
H. sapiens	121072	ORF frame 3	Synthetic
H. sapiens	112174	ORF frame 3	Synthetic
H. sapiens	78373	ORF frame 2	Synthetic

Supplementary Table 8. Natural test set sequences.

Organism	Internal ID	Link to Sequence
E. coli	Test_0001	http://ecocyc.org/ECOLI/sequence?type=GENE&object=G7176
E. coli	Test_0002	http://ecocyc.org/ECOLI/sequence-rc?type=GENE&object=EG11972
E. coli	Test_0003	http://ecocyc.org/ECOLI/sequence-rc?type=GENE&object=G7554
E. coli	Test_0004	http://ecocyc.org/ECOLI/sequence?type=GENE&object=EG11212
E. coli	Test_0005	http://ecocyc.org/ECOLI/sequence?type=GENE&object=EG10894
E. coli	Test_0006	http://ecocyc.org/ECOLI/sequence?type=GENE&object=EG11373
E. coli	Test_0007	http://ecocyc.org/ECOLI/sequence?type=GENE&object=EG10589
E. coli	Test_0008	http://ecocyc.org/ECOLI/sequence-rc?type=GENE&object=G7595
E. coli	Test_0009	http://ecocyc.org/ECOLI/sequence-rc?type=GENE&object=EG10109
E. coli	Test_0010	http://ecocyc.org/ECOLI/sequence?type=GENE&object=G6877
E. coli	Test_0011	http://ecocyc.org/ECOLI/sequence-rc?type=GENE&object=EG10757
E. coli	Test_0012	http://ecocyc.org/ECOLI/sequence-rc?type=GENE&object=EG11538
E. coli	Test_0013	http://ecocyc.org/ECOLI/sequence-rc?type=GENE&object=EG11241
E. coli	Test_0014	http://ecocyc.org/ECOLI/sequence-rc?type=GENE&object=G7028
E. coli	Test_0015	http://ecocyc.org/ECOLI/sequence-rc?type=GENE&object=EG10856
E. coli	Test_0016	http://ecocyc.org/ECOLI/sequence-rc?type=GENE&object=G6326
E. coli	Test_0017	http://ecocyc.org/ECOLI/sequence-rc?type=GENE&object=G6456
E. coli	Test_0018	http://ecocyc.org/ECOLI/sequence?type=GENE&object=EG10620
E. coli	Test_0019	http://ecocyc.org/ECOLI/sequence?type=GENE&object=G6320
E. coli	Test_0020	http://ecocyc.org/ECOLI/sequence-rc?type=GENE&object=EG10782
E. coli	Test_0021	http://ecocyc.org/ECOLI/sequence?type=GENE&object=G6573
E. coli	Test_0022	http://ecocyc.org/ECOLI/sequence-rc?type=GENE&object=G7414
E. coli	Test_0023	http://ecocyc.org/ECOLI/sequence-rc?type=GENE&object=EG12244
E. coli	Test_0024	http://ecocyc.org/ECOLI/sequence?type=GENE&object=EG10618
E. coli	Test_0025	http://ecocyc.org/ECOLI/sequence?type=GENE&object=EG11734
S. cerevisiae	Test_0026	http://www.yeastgenome.org/locus/S000000094/sequence
S. cerevisiae	Test_0027	http://www.yeastgenome.org/locus/S000000314/sequence
S. cerevisiae	Test_0028	http://www.yeastgenome.org/locus/S000000687/sequence
S. cerevisiae	Test_0029	http://www.yeastgenome.org/locus/S000002441/sequence
S. cerevisiae	Test_0030	http://www.yeastgenome.org/locus/S000002851/sequence
S. cerevisiae	Test_0031	http://www.yeastgenome.org/locus/S000000737/sequence
S. cerevisiae	Test_0032	http://www.yeastgenome.org/locus/S000000927/sequence
S. cerevisiae	Test_0033	http://www.yeastgenome.org/locus/S000001873/sequence
S. cerevisiae	Test_0034	http://www.yeastgenome.org/locus/S000001906/sequence
S. cerevisiae	Test_0035	http://www.yeastgenome.org/locus/S000003416/sequence
S. cerevisiae	Test_0036	http://www.yeastgenome.org/locus/S000003024/sequence
S. cerevisiae	Test_0037	http://www.yeastgenome.org/locus/S000001133/sequence
S. cerevisiae	Test_0038	http://www.yeastgenome.org/locus/S000001128/sequence
S. cerevisiae	Test_0039	http://www.yeastgenome.org/locus/S000001381/sequence

S. cerevisiae	Test_0040	http://www.yeastgenome.org/locus/S000001356/sequence
S. cerevisiae	Test_0041	http://www.yeastgenome.org/locus/S000003913/sequence
S. cerevisiae	Test_0042	http://www.yeastgenome.org/locus/S000003792/sequence
S. cerevisiae	Test_0043	http://www.yeastgenome.org/locus/S000001599/sequence
S. cerevisiae	Test_0044	http://www.yeastgenome.org/locus/S000001586/sequence
S. cerevisiae	Test_0045	http://www.yeastgenome.org/locus/S000004145/sequence
S. cerevisiae	Test_0046	http://www.yeastgenome.org/locus/S000004404/sequence
S. cerevisiae	Test_0047	http://www.yeastgenome.org/locus/S000004628/sequence
S. cerevisiae	Test_0048	http://www.yeastgenome.org/locus/S000005056/sequence
S. cerevisiae	Test_0049	http://www.yeastgenome.org/locus/S000005572/sequence
S. cerevisiae	Test_0050	http://www.yeastgenome.org/locus/S000006036/sequence
H. sapiens	Test_0051	http://genomics.senescence.info/genes/seq.php?id=243&type=orf
H. sapiens	Test_0052	http://genomics.senescence.info/genes/seq.php?id=127&type=orf
H. sapiens	Test_0053	http://genomics.senescence.info/genes/seq.php?id=158&type=orf
H. sapiens	Test_0054	http://genomics.senescence.info/genes/seq.php?id=221&type=orf
H. sapiens	Test_0055	http://genomics.senescence.info/genes/seq.php?id=209&type=orf
H. sapiens	Test_0056	http://genomics.senescence.info/genes/seq.php?id=90&type=orf
H. sapiens	Test_0057	http://genomics.senescence.info/genes/seq.php?id=39&type=orf
H. sapiens	Test_0058	http://genomics.senescence.info/genes/seq.php?id=44&type=orf
H. sapiens	Test_0059	http://genomics.senescence.info/genes/seq.php?id=43&type=orf
H. sapiens	Test_0060	http://genomics.senescence.info/genes/seq.php?id=276&type=orf
H. sapiens	Test_0061	http://genomics.senescence.info/genes/seq.php?id=307&type=orf
H. sapiens	Test_0062	http://genomics.senescence.info/genes/seq.php?id=283&type=orf
H. sapiens	Test_0063	http://genomics.senescence.info/genes/seq.php?id=82&type=orf
H. sapiens	Test_0064	http://genomics.senescence.info/genes/seq.php?id=20&type=orf
H. sapiens	Test_0065	http://genomics.senescence.info/genes/seq.php?id=219&type=orf
H. sapiens	Test_0066	http://genomics.senescence.info/genes/seq.php?id=31&type=orf
H. sapiens	Test_0067	http://genomics.senescence.info/genes/seq.php?id=37&type=orf
H. sapiens	Test_0068	http://genomics.senescence.info/genes/seq.php?id=229&type=orf
H. sapiens	Test_0069	http://genomics.senescence.info/genes/seq.php?id=75&type=orf
H. sapiens	Test_0070	http://genomics.senescence.info/genes/seq.php?id=24&type=orf
H. sapiens	Test_0071	http://genomics.senescence.info/genes/seq.php?id=254&type=orf
H. sapiens	Test_0072	http://genomics.senescence.info/genes/seq.php?id=60&type=orf
H. sapiens	Test_0073	http://genomics.senescence.info/genes/seq.php?id=248&type=orf
H. sapiens	Test_0074	http://genomics.senescence.info/genes/seq.php?id=162&type=orf
H. sapiens	Test_0075	http://genomics.senescence.info/genes/seq.php?id=113&type=orf

Supplementary Table 9. Synthetic test sequences from iGEM directory.

Name	Internal ID	Classification
BBa_K592009	Test_0076	Synthetic
BBa_K592011	Test_0077	Synthetic
BBa_K1122007	Test_0078	Synthetic
BBa_K1033902	Test_0079	Synthetic
BBa_K1033906	Test_0080	Synthetic
BBa_K1033916	Test_0081	Synthetic
BBa_K1033910	Test_0082	Synthetic
BBa_K592010	Test_0083*	Natural
BBa_K864401	Test_0084	Synthetic
BBa_K1033919	Test_0085	Synthetic
BBa_K1033932	Test_0086	Synthetic
BBa_J97001	Test_0087	Synthetic
BBa_J97000	Test_0088	Synthetic
BBa_I742107	Test_0089*	Natural
BBa_K1686046	Test_0090	Synthetic
BBa_K1319004	Test_0091	Synthetic
BBa_K1907001	Test_0092	Synthetic
BBa_K1907002	Test_0093	Synthetic
BBa_M1436	Test_0094	Synthetic
BBa_M1437	Test_0095	Synthetic
BBa_K1758313	Test_0096	Synthetic
BBa_K1758310	Test_0097	Synthetic
BBa_K1319004	Test_0098	Synthetic
BBa_K1319003	Test_0099	Synthetic
BBa_K1506002	Test_0100	Synthetic
BBa_K1506000	Test_0101	Synthetic
BBa_K1506001	Test_0102	Synthetic
BBa_K2148013	Test_0103	Synthetic
BBa_K325902	Test_0104	Synthetic
BBa_K325903	Test_0105	Synthetic
BBa_K2165000	Test_0106	Synthetic
BBa_K2165001	Test_0107	Synthetic
BBa_K315040	Test_0108**	Mostly Natural
BBa_K382020	Test_0109	Synthetic
BBa_K382021	Test_0110	Synthetic
BBa_K1314017	Test_0111	Synthetic
BBa_K801080	Test_0112	Synthetic
BBa_Y00029	Test_0113	Synthetic
BBa_K1959000	Test_0114*	Natural

BBa_K1959001	Test_0115	Synthetic
BBa_K1959002	Test_0116	Synthetic
BBa_K1959003	Test_0117	Synthetic
BBa_K1717171	Test_0118	Synthetic
BBa_K1717172	Test_0119	Synthetic
BBa_K844004	Test_0120	Synthetic

*In cases marked by an asterisk above, the sequence was determined to be natural upon closer examination.

**In the case marked by a double asterisks above, it appears only one quarter of the gene was codon optimized and thus the sequence is mostly 100% identical to a naturally occurring sequence annotated with the same function/origin.

Supplementary Table 10. Synthetic *S. cerevisiae* sequences from literature.

Name	Internal ID	Reference
yPbSalSyn	Test_0121	Galanie et al
yEcCFS1-83-yPbSalSyn92-504	Test_0122	Galanie et al
yPbDRS-DRR (Pbr.89405)	Test_0123	Galanie et al
yPsDRS-DRR(Pso.2062398)	Test_0124	Galanie et al
yPbDRS-DRR (Pbr.12180)	Test_0125	Galanie et al
yPbDRS-DRR (Pbr.4328)	Test_0126	Galanie et al
yPbSalAT-1	Test_0127	Galanie et al
yPbSalAT-2	Test_0128	Galanie et al
yPoSalAT	Test_0129	Galanie et al
yPsAT1	Test_0130	Galanie et al
eGFP control table (CT)	Test_0131	Lanza et al
eGFP high expression table (HT)	Test_0132	Lanza et al
eGFP control matrix 1 (C1)	Test_0133	Lanza et al
eGFP control matrix 2 (C2)	Test_0134	Lanza et al
eGFP control matrix 3 (C3)	Test_0135	Lanza et al
eGFP high expression matrix 1 (H1)	Test_0136	Lanza et al
eGFP high expression matrix 2 (H2)	Test_0137	Lanza et al
eGFP high expression matrix 3 (H3)	Test_0138	Lanza et al
CatA Blue Heron	Test_0139	Lanza et al
CatA control 1 (C1)	Test_0140	Lanza et al
CatA control 2 (C2)	Test_0141	Lanza et al
CatA control 3 (C3)	Test_0142	Lanza et al
CatA stationary 1 (S1)	Test_0143	Lanza et al
CatA stationary 2 (S2)	Test_0144	Lanza et al
CatA stationary 3 (S3)	Test_0145	Lanza et al
CatA high expression 1 (H1)	Test_0146	Lanza et al
CatA high expression 2 (H2)	Test_0147	Lanza et al

Supplementary Table 11. Synthetic sequences for expression in *H. sapiens*.

Name or Link to Sequence	Internal ID
Humanized aequorin	Test_0148
Preferred aequorin	Test_0149
Preferred clytin II	Test_0150
Preferred Gaussia princeps luciferase	Test_0151
Humanized OG luciferase	Test_0152
Preferred OG luciferase	Test_0153
Humanized RR luciferase	Test_0154
Preferred RR luciferase	Test_0155
Preferred PP luciferase	Test_0156
Preferred Lc luciferase	Test_0157
https://www.ncbi.nlm.nih.gov/nuccore/FW361641.1	Test_0158
https://www.ncbi.nlm.nih.gov/nuccore/DL143697.1	Test_0159
https://www.ncbi.nlm.nih.gov/nuccore/JF727882.1	Test_0160
https://www.ncbi.nlm.nih.gov/nuccore/HZ783837.1	Test_0161
https://www.ncbi.nlm.nih.gov/nuccore/DQ421322.1	Test_0162
https://www.ncbi.nlm.nih.gov/nuccore/DQ421318.1	Test_0163
https://www.ncbi.nlm.nih.gov/nuccore/BD493373.1	Test_0164
https://www.ncbi.nlm.nih.gov/nuccore/AY995800.1	Test_0165
https://www.ncbi.nlm.nih.gov/nuccore/DQ667595.1	Test_0166
https://www.ncbi.nlm.nih.gov/nuccore/EF415639.1	Test_0167
https://www.ncbi.nlm.nih.gov/nuccore/EF415638.1	Test_0168
https://www.ncbi.nlm.nih.gov/nuccore/LQ263568.1	Test_0169
https://www.ncbi.nlm.nih.gov/nuccore/LQ468667.1	Test_0170
https://www.ncbi.nlm.nih.gov/nuccore/LQ263569.1	Test_0171
https://www.ncbi.nlm.nih.gov/nuccore/KF420121.1	Test_0172
https://www.ncbi.nlm.nih.gov/nuccore/HB649493.1	Test_0173

Supplementary Table 12. Parameter sensitivity analysis on %ID and %QCov

Legend

0%	90%	100%

		% Identity Cutoff					
		70%	75%	80%	85%	90%	95%
% Query Coverage Cutoff	70%	75%	90%	97%	98%	95%	93%
	75%	75%	90%	97%	98%	95%	93%
	80%	75%	90%	97%	98%	95%	93%
	85%	75%	90%	97%	98%	95%	93%
	90%	75%	90%	97%	98%	95%	93%
	95%	84%	91%	97%	98%	95%	93%

Supplementary Table 13. Simplification of Addgene expression data entries.

Original Addgene Expression Data	Categories
AAV	Mammalian
AAV, Cre/Lox	Mammalian
AAV, Cre/Lox, Other	Mammalian
AAV, Other	Mammalian
AAV, Other, Unspecified	Mammalian
Adenoviral	Mammalian
Adenoviral, Other	Mammalian
Bacterial Expression	Bacterial
Bacterial Expression, Cre/Lox	Bacterial
Bacterial Expression, Cre/Lox, Other	Bacterial
Bacterial Expression, CRISPR	Bacterial
Bacterial Expression, CRISPR, Other	Bacterial
Bacterial Expression, CRISPR, Synthetic Biology	Bacterial
Bacterial Expression, Insect Expression	Insect
Bacterial Expression, Insect Expression, Other	Insect
Bacterial Expression, Luciferase	Bacterial
Bacterial Expression, Luciferase, Other	Bacterial
Bacterial Expression, Luciferase, Synthetic Biology, Other	Bacterial
Bacterial Expression, Other	Bacterial
Bacterial Expression, Other, Unspecified	Bacterial
Bacterial Expression, Plant Expression	Plant
Bacterial Expression, Retroviral	Mammalian
Bacterial Expression, RNAi	Bacterial
Bacterial Expression, Synthetic Biology	Bacterial
Bacterial Expression, Synthetic Biology, Other	Bacterial
Bacterial Expression, TALEN	Bacterial
Bacterial Expression, Unspecified	Bacterial
Bacterial Expression, Worm Expression	Worm
Bacterial Expression, Yeast Expression	Yeast
Bacterial Expression, Yeast Expression, Cre/Lox	Yeast
Bacterial Expression, Yeast Expression, CRISPR	Yeast
Bacterial Expression, Yeast Expression, CRISPR, Synthetic Biology	Yeast
Bacterial Expression, Yeast Expression, Other	Yeast
Bacterial Expression, Yeast Expression, Synthetic Biology	Yeast
Bacterial Expression, Yeast Expression, Synthetic Biology, Other	Yeast
Cre/Lox	Unspecified
Cre/Lox, Other	Unspecified
CRISPR	Unspecified
CRISPR, Other	Unspecified
CRISPR, Synthetic Biology	Unspecified

CRISPR, Unspecified	Unspecified
Insect Expression	Insect
Insect Expression, Cre/Lox	Insect
Insect Expression, CRISPR	Insect
Insect Expression, CRISPR, Other	Insect
Insect Expression, CRISPR, Synthetic Biology, Other	Insect
Insect Expression, Luciferase	Insect
Insect Expression, Luciferase, Other	Insect
Insect Expression, Other	Insect
Insect Expression, Retroviral	Mammalian
Insect Expression, RNAi	Insect
Insect Expression, TALEN	Insect
Lentiviral	Mammalian
Lentiviral, Cre/Lox	Mammalian
Lentiviral, Cre/Lox, CRISPR	Mammalian
Lentiviral, Cre/Lox, Luciferase	Mammalian
Lentiviral, CRISPR	Mammalian
Lentiviral, Luciferase	Mammalian
Lentiviral, Other	Mammalian
Lentiviral, RNAi	Mammalian
Lentiviral, Synthetic Biology	Mammalian
Luciferase	Unspecified
Luciferase, Other	Unspecified
Luciferase, Synthetic Biology	Unspecified
Mammalian Expression	Mammalian
Mammalian Expression	Mammalian
Mammalian Expression, AAV	Mammalian
Mammalian Expression, AAV, Cre/Lox	Mammalian
Mammalian Expression, AAV, Cre/Lox, Other	Mammalian
Mammalian Expression, AAV, Cre/Lox, Synthetic Biology	Mammalian
Mammalian Expression, AAV, CRISPR	Mammalian
Mammalian Expression, AAV, Other	Mammalian
Mammalian Expression, AAV, RNAi	Mammalian
Mammalian Expression, AAV, RNAi, Cre/Lox	Mammalian
Mammalian Expression, AAV, RNAi, Other	Mammalian
Mammalian Expression, AAV, Synthetic Biology	Mammalian
Mammalian Expression, AAV, TALEN	Mammalian
Mammalian Expression, Adenoviral	Mammalian
Mammalian Expression, Bacterial Expression	Mammalian
Mammalian Expression, Bacterial Expression, CRISPR	Mammalian
Mammalian Expression, Bacterial Expression, Insect Expression	Mammalian
Mammalian Expression, Bacterial Expression, Lentiviral	Mammalian
Mammalian Expression, Bacterial Expression, Lentiviral, Other	Mammalian

Mammalian Expression, Bacterial Expression, Luciferase	Mammalian
Mammalian Expression, Bacterial Expression, Mouse Targeting	Mammalian
Mammalian Expression, Bacterial Expression, Mouse Targeting, Cre/Lox	Mammalian
Mammalian Expression, Bacterial Expression, Mouse Targeting, Other	Mammalian
Mammalian Expression, Bacterial Expression, Retroviral	Mammalian
Mammalian Expression, Bacterial Expression, Worm Expression, Other	Mammalian
Mammalian Expression, Bacterial Expression, Yeast Expression	Mammalian
Mammalian Expression, Bacterial Expression, Yeast Expression, Insect Expression, Other	Mammalian
Mammalian Expression, Bacterial Expression, Yeast Expression, Insect Expression, Plant Expression, Synthetic Biology	Mammalian
Mammalian Expression, Bacterial Expression, Yeast Expression, Lentiviral	Mammalian
Mammalian Expression, Cre/Lox	Mammalian
Mammalian Expression, Cre/Lox, Other	Mammalian
Mammalian Expression, CRISPR	Mammalian
Mammalian Expression, CRISPR, Other	Mammalian
Mammalian Expression, CRISPR, Synthetic Biology	Mammalian
Mammalian Expression, CRISPR, Synthetic Biology, Other	Mammalian
Mammalian Expression, CRISPR, TALEN	Mammalian
Mammalian Expression, CRISPR, TALEN, Other	Mammalian
Mammalian Expression, Insect Expression, Luciferase	Mammalian
Mammalian Expression, Lentiviral	Mammalian
Mammalian Expression, Lentiviral, Cre/Lox	Mammalian
Mammalian Expression, Lentiviral, CRISPR	Mammalian
Mammalian Expression, Lentiviral, CRISPR, Synthetic Biology	Mammalian
Mammalian Expression, Lentiviral, Luciferase	Mammalian
Mammalian Expression, Lentiviral, Other	Mammalian
Mammalian Expression, Lentiviral, Retroviral	Mammalian
Mammalian Expression, Lentiviral, RNAi	Mammalian
Mammalian Expression, Lentiviral, RNAi, Cre/Lox	Mammalian
Mammalian Expression, Lentiviral, RNAi, Other	Mammalian
Mammalian Expression, Lentiviral, Synthetic Biology	Mammalian
Mammalian Expression, Lentiviral, TALEN	Mammalian
Mammalian Expression, Luciferase	Mammalian
Mammalian Expression, Luciferase, Other	Mammalian
Mammalian Expression, Mouse Targeting	Mammalian
Mammalian Expression, Mouse Targeting, AAV	Mammalian
Mammalian Expression, Mouse Targeting, AAV, Cre/Lox	Mammalian
Mammalian Expression, Mouse Targeting, AAV, Cre/Lox, CRISPR	Mammalian
Mammalian Expression, Mouse Targeting, AAV, Cre/Lox, CRISPR, Luciferase	Mammalian
Mammalian Expression, Mouse Targeting, AAV, CRISPR	Mammalian
Mammalian Expression, Mouse Targeting, AAV, RNAi, Cre/Lox	Mammalian
Mammalian Expression, Mouse Targeting, Cre/Lox	Mammalian
Mammalian Expression, Mouse Targeting, Cre/Lox, CRISPR	Mammalian

Mammalian Expression, Mouse Targeting, Cre/Lox, Other	Mammalian
Mammalian Expression, Mouse Targeting, CRISPR, TALEN	Mammalian
Mammalian Expression, Mouse Targeting, Lentiviral, Cre/Lox, CRISPR	Mammalian
Mammalian Expression, Mouse Targeting, Other	Mammalian
Mammalian Expression, Mouse Targeting, Retroviral	Mammalian
Mammalian Expression, Mouse Targeting, RNAi, Cre/Lox	Mammalian
Mammalian Expression, Mouse Targeting, TALEN	Mammalian
Mammalian Expression, Other	Mammalian
Mammalian Expression, Retroviral	Mammalian
Mammalian Expression, Retroviral, Cre/Lox	Mammalian
Mammalian Expression, Retroviral, Cre/Lox, Other	Mammalian
Mammalian Expression, Retroviral, CRISPR	Mammalian
Mammalian Expression, Retroviral, CRISPR, Other	Mammalian
Mammalian Expression, Retroviral, Luciferase	Mammalian
Mammalian Expression, Retroviral, Other	Mammalian
Mammalian Expression, Retroviral, RNAi	Mammalian
Mammalian Expression, Retroviral, RNAi, Cre/Lox	Mammalian
Mammalian Expression, Retroviral, RNAi, Other	Mammalian
Mammalian Expression, Retroviral, Synthetic Biology	Mammalian
Mammalian Expression, Retroviral, TALEN	Mammalian
Mammalian Expression, RNAi	Mammalian
Mammalian Expression, RNAi, Cre/Lox	Mammalian
Mammalian Expression, RNAi, Other	Mammalian
Mammalian Expression, Synthetic Biology	Mammalian
Mammalian Expression, Synthetic Biology, Other	Mammalian
Mammalian Expression, TALEN	Mammalian
Mammalian Expression, TALEN, Other	Mammalian
Mammalian Expression, Worm Expression, Other	Mammalian
Mammalian Expression, Yeast Expression	Mammalian
Mouse Targeting	Mammalian
Mouse Targeting, Cre/Lox	Mammalian
Mouse Targeting, Other	Mammalian
Mouse Targeting, RNAi	Mammalian
Mouse Targeting, Synthetic Biology	Mammalian
N/A	Unspecified
Other	Unspecified
Plant Expression	Plant
Plant Expression, Cre/Lox	Plant
Plant Expression, CRISPR	Plant
Plant Expression, CRISPR, Other	Plant
Plant Expression, CRISPR, Synthetic Biology	Plant
Plant Expression, Luciferase, Synthetic Biology	Plant
Plant Expression, Other	Plant

Plant Expression, Synthetic Biology	Plant
Plant Expression, TALEN	Plant
Retroviral	Mammalian
Retroviral, Luciferase	Mammalian
Retroviral, Other	Mammalian
Retroviral, RNAi	Mammalian
Retroviral, RNAi, Other	Mammalian
RNAi	Unspecified
RNAi, Other	Unspecified
Synthetic Biology	Unspecified
Synthetic Biology, Other	Unspecified
TALEN	Unspecified
TALEN, Other	Unspecified
Unspecified	Unspecified
Worm Expression	Worm
Worm Expression, Cre/Lox	Worm
Worm Expression, Cre/Lox, CRISPR	Worm
Worm Expression, Cre/Lox, Other	Worm
Worm Expression, CRISPR	Worm
Worm Expression, CRISPR, Other	Worm
Worm Expression, Other	Worm
Worm Expression, RNAi	Worm
Worm Expression, RNAi, Other	Worm
Yeast Expression	Yeast
Yeast Expression, Cre/Lox	Yeast
Yeast Expression, Cre/Lox, Other	Yeast
Yeast Expression, CRISPR	Yeast
Yeast Expression, CRISPR, Other	Yeast
Yeast Expression, CRISPR, Synthetic Biology	Yeast
Yeast Expression, Other	Yeast
Yeast Expression, Synthetic Biology	Yeast
Yeast Expression, Synthetic Biology, Other	Yeast
Yeast Expression, TALEN	Yeast
#N/A	Unspecified

Supplementary Table 14. Regression result for genetic distance analysis excluding sequences identified as CRISPR-Cas9. Values in parentheses are standard errors, stars indicate t-test statistical significance.

	Dependent variable:			
	Genetic Distance	Genetic Distance	Cross Kingdom	Cross Kingdom
	OLS	OLS	OLS	Logit
	(5)	(6)	(7)	(8)
Constant	0.488***	0.599***	0.574***	0.521***
	(0.006)	(0.009)	(0.007)	(0.038)
Synthetic	0.059***	-0.048	-0.016	-0.283***
	(0.020)	(0.030)	(0.024)	(0.102)
Gene Length [kb]		-0.116***	-0.107***	-0.697***
		(0.007)	(0.005)	(0.039)
Gene Length [kb] * Synthetic		0.114***	0.075***	0.569***
		(0.016)	(0.012)	(0.061)
Observations	14,080	14,080	14,080	14,080
R2	0.000	0.019	0.027	
Adjusted R2	0.000	0.019	0.027	
Log Likelihood				-9493.344
F statistic	8.70	92.64	130.31	

*p<0.1; **p<0.05; ***p<0.01

Supplementary Methods

Training set construction

Natural *E. coli* sequences and synthetic sequences that were codon optimized for *E. coli* expression were obtained from Addgene based on keyword searches for “synthetic” or “codon-optimized” and were verified from the plasmid description or how “insert” genes were sourced (often described in the “Methods” section of the associated publication). These genes are shown in Supplementary Table 4, where the sequence number follows the website link www.addgene.org/browse/sequence/. For yeast (*S. cerevisiae*), 19 natural sequences were obtained randomly from the Yeast Genome Browser (www.yeastgenome.org/browse/). Synthetic sequences codon optimized for yeast expression were obtained in two ways. First, 20 native sequences were entered into the online IDT codon optimization tool (www.idtdna.com/CodonOpt) and optimized with "Saccharomyces cerevisiae" selected as the organism. Second, four additional synthetic sequences for yeast expression were obtained from Addgene and are shown in Supplementary Tables 5 and 6. For human (*H. sapiens*), two natural sequences were obtained by selecting genes from the Human Ageing Genomic Resources "GenAge" Database (genomics.senescence.info/genes/allgenes.php) and then by gathering the corresponding cDNA from EMBL-EBI (www.ebi.ac.uk/). Synthetic sequences codon optimized for human expression were obtained from Addgene are shown in Supplementary Table 7. In addition, five natural sequences from a relatively rare organism (*Nocardia iowensis*) were obtained from GenBank and used to verify the presence of rare sequences in the RefSeq Database.

Test set construction

We constructed the test set with natural sequences from *E. coli*, *S. cerevisiae*, and *H. sapiens*. We chose 25 natural *E. coli* sequences using randomly generated positions in the *E. coli* MG1655 genome (ecocyc.org). We also chose 25 natural *S. cerevisiae* sequences using randomly generated positions distributed across the 16 chromosomes (www.yeastgenome.org). We chose 25 natural *H. sapiens* sequences again using the Human Ageing Database but this time starting from genes beginning with the letter “M”. These natural test set sequences can be found in Supplementary Table 8.

For synthetic sequences in the test set, we first considered using the International Genetically Engineered Machine (iGEM) parts database (also known as the Registry of Standard Biological Parts, parts.igem.org). Supplementary Table 9 lists the 45 synthetic iGEM parts sourced, which are primarily codon-optimized for *E. coli*.

We obtained 27 synthetic sequences codon optimized for expression in *S. cerevisiae* from two recent publications in the scientific literature[2,3]. These sequences are shown in Supplementary Table 10.

Finally, we extracted sequences codon optimized for expression in *H. sapiens* from two sources. The first source was a recent publication on mammalian codon-optimization that provided ten sequences[4]. The second source was the NCBI database using the search term "human codon optimized" and subsequently filtering by "synthetic construct." These sequences are shown in Supplementary Table 11.

Code

The following R code was used for the codon optimization simulation:

```
### Synthesis Cost Project - Codon Optimization Simulation
### Code by Neil Thompson
### Data from Aditya Kunjapur
### June 6, 2017


### Functions

Populate.Organism.Data       <- function(directory){
  ### Creates data frames for each organism, based on the data on
  ### amino acid and codon frequency from Aditya

  files = list.files(directory)

  organism.data = list()

  for(file in files){
    organism <- strsplit(file, split=".csv")[[1]]

    # import data
    organism.table <- read.csv(
                            file = paste(directory,file,sep=""),
                            header = FALSE,
                            blank.lines.skip = TRUE)

    # names fields
    colnames(organism.table)  <- c("codon",
                             "amino.acid",
                             "freq.for.amino.acid",
                             "freq.in.all.codons",
                             "raw.count")
    # delete empty rows
    organism.table <- organism.table[organism.table$codon != "" ,]

    # keep organism name with table
    attributes(organism.table)$organism.name <- organism
```

```
    organism.data[[organism]] <- organism.table

  }

  return(organism.data)
}

Populate.Amino.Acid.And.Codon.Lists     <- function(organism.data){
  ### Fills in reference tables for the amino acids and codons
  ### for each organism, based on the raw data from Aditya

  amino.acid.and.codon.data <- list(amino.acid.list = list(),
                                    codon.list      = list())


  # get list of all amino acids and codons used across all organisms
  all.amino.acids = levels(unlist(lapply(organism.data, function(x) unique(x$amino.acid))))
  all.amino.acids = all.amino.acids[all.amino.acids != ""]

  all.codons      = levels(unlist(lapply(organism.data, function(x) unique(x$codon))))
  all.codons      = all.codons[all.codons != ""]

  ## build amino acid and codon list
  for(organism in names(organism.data)){

    ## Build data frame for amino acids
    amino.acids <- aggregate(x   = organism.data[[organism]]$freq.in.all.codons,
                             by  = list(organism.data[[organism]]$amino.acid),
                             FUN = "sum")

    colnames(amino.acids) <- c("name", "prob")

    missing.amino.acids   <- setdiff(all.amino.acids, amino.acids[[1]])

    if(!identical(missing.amino.acids,character(0))){
      amino.acids <- rbind(amino.acids,
                           data.frame(name = missing.amino.acids, prob = 0))
    }

    # normalize to address rounding issues
    amino.acids$prob <- amino.acids$prob * 1/sum(amino.acids$prob)


    # add tag to data frame
    attributes(amino.acids)$organism.name = organism

    # save
    amino.acid.and.codon.data$amino.acid.list[[organism]] <- amino.acids


    ## Build list for codons
    codons <- organism.data[[organism]][c("codon", "amino.acid", "freq.in.all.codons")]
    colnames(codons) <- c("codon", "name", "prob")

    if(!identical(missing.amino.acids,character(0))){
      codons <- rbind(codons,
                      data.frame(codon = "???", name = missing.amino.acids, prob = 1))
    }

    # build one data.frame for each amino acid
    codons <- split(codons, codons$name)
    codons <- codons[names(codons) != ""]

    # normalize to address rounding issues
    for(aa in names(codons)){
      codons[[aa]]$prob = codons[[aa]]$prob * 1/sum(codons[[aa]]$prob)
    }

    # add tag to data frame
    attributes(codons)$organism.name = organism
```

```
    # save
    amino.acid.and.codon.data$codon.list[[organism]] <- codons

  }

  return(amino.acid.and.codon.data)
}




Generate.Amino.Acid.Sequence <- function(amino.acids, sequence.length){
  # Generates a random amino acid sequence from the frequencies given

  amino.acid.sequence <- sample(x       = amino.acids$name,
                                size    = sequence.length,
                                replace = TRUE,
                                prob    = amino.acids$prob)
  return(amino.acid.sequence)
}



Generate.Codon.Sequence      <- function(amino.acid.seq, codon.table){
  # Generates a random codon sequence based on an amino acid sequence based on the codon
frequency of the organism

  # sequence
  codon.seq = rep(NA, length(amino.acid.seq))

  # do weighted sampling
  for(j in 1:length(amino.acid.seq)){
    amino.acid        = as.character(amino.acid.seq[j])
    new.codon         = sample(x       = as.character(codon.table[[amino.acid]]$codon),
                               size    = 1,
                               replace = TRUE,
                               prob    = as.character(codon.table[[amino.acid]]$prob))
    codon.seq[j] = new.codon
  }
  return(codon.seq)
}


Compare.Codon.Sequences      <- function(codon.seq.1, codon.seq.2){
  # Calculates the similarity of two sequences at the BASE level
  seq.1 = unlist(strsplit(codon.seq.1, split = ""))
  seq.2 = unlist(strsplit(codon.seq.2, split = ""))

  codon.similarity <- sum(seq.1 == seq.2)/length(seq.1)

  return(codon.similarity)
}


### Simulation

# Parameters and System Settings
directory       = "C:/Users/neil_t/Dropbox\ (MIT)/Synthesis\ Costs/Code/Simulation\ for\
sequence\ identity/Codon\ Usage\ CSV/"
num.repetitions = 1000
sequence.length = 1000

# Populate data on organisms
organism.data   <- Populate.Organism.Data(directory)
organisms       <- names(organism.data)

# construct list
amino.acid.and.codon.data <- Populate.Amino.Acid.And.Codon.Lists(organism.data)
amino.acid.list           <- amino.acid.and.codon.data[[1]]
codon.list                <- amino.acid.and.codon.data[[2]]
```

```
# Data structures
mean.similarity     = matrix(nrow     = length(organisms),
                             ncol     = length(organisms),
                             dimnames = list(organisms, organisms))
stdev.similarity     = matrix(nrow    = length(organisms),
                             ncol     = length(organisms),
                             dimnames = list(organisms, organisms))
all.outcomes        = list()


for(source.organism in organisms){
  for(expression.organism in organisms){

    simulation.outcomes   <- rep(NA, num.repetitions)

    for(i in 1:num.repetitions){

      ## Generate Sequences for comparison

      # Generate amino acid sequence from the source organism
      amino.acid.seq        <- Generate.Amino.Acid.Sequence(
                                   amino.acids     = amino.acid.list[[source.organism]],
                                   sequence.length = sequence.length)

      # Generate a codon sequence for the amino acid sequence consistent with the source organism
      source.codon.seq      <- Generate.Codon.Sequence(
                                   amino.acid.seq  = amino.acid.seq,
                                   codon.table     = codon.list[[source.organism]])

      # Generate a codon sequence for the amino acid sequence consistent with the expression
organism
      expression.codon.seq <- Generate.Codon.Sequence(
                                   amino.acid.seq  = amino.acid.seq,
                                   codon.table     = codon.list[[expression.organism]])

      ## Calculate the similarity between the codon sequences and save
      simulation.outcomes[i] <- Compare.Codon.Sequences(source.codon.seq, expression.codon.seq)

    }

    # Save Simulation Outcomes
    all.outcomes[[paste(source.organism, expression.organism, sep=" : ")]] <- simulation.outcomes
    mean.similarity[source.organism, expression.organism] <- mean(simulation.outcomes)
    stdev.similarity[source.organism, expression.organism] <- sd(simulation.outcomes)

  }
}

## Boxplot of similarity for expression in humans (plots)

# create data frame
source.organisms <- c()
identity.data <- c()

expression.organism = "H sapiens"

for(source.organism in organisms){
  source.organisms = append(source.organisms, rep(source.organism,
length(all.outcomes[[paste(source.organism, expression.organism, sep=" : ")]])))
  identity.data = append(identity.data,all.outcomes[[paste(source.organism, expression.organism,
sep=" : ")]])
}

boxplot.data2 <- data.frame(source.organisms, identity.data)

par(mar=c(10,5,1,1))
boxplot(boxplot.data2$identity.data ~ boxplot.data2$source.organisms,
        col  = "grey",
        ylim = c(0.6,0.9),
        ylab = "% Identity",
```

```
        yaxt = "n",
        las  = 2)
axis(2, at=pretty(boxplot.data2$identity.data),
lab=paste0(pretty(boxplot.data2$identity.data)*100,"%"), las=TRUE, ylim = c(0.6,0.9))
abline(h=0.85, lty=3)

# Density plot for each expression organism by source organism
densityplot.data            <- data.frame(matrix(nrow=length(organisms), ncol=100, data=0))
row.names(densityplot.data) <- organisms
names(densityplot.data)     <- as.character((1:100)/100)

for(expression.organism in organisms){
  expression.data <- c()

  # append data from all source organisms
  for(source.organism in organisms){
    expression.data <- append(expression.data, all.outcomes[[paste(source.organism,
expression.organism, sep=" : ")]])
  }

  # round data
  rounded.data = round(expression.data, digits=2)
  tab.data = as.data.frame(table(rounded.data), stringsAsFactors = F)
  densityplot.data[expression.organism, tab.data$rounded.data] <- tab.data$Freq
}

write.csv(densityplot.data, file = "C:/Users/neil_t/Dropbox\ (MIT)/Synthesis\
Costs/Code/MonteCarloDensities.csv")

save.image(paste("C:/Users/neil_t/Dropbox\ (MIT)/Synthesis\ Costs/Code/Codon\ Optimization\
Simulation\ Results", sequence.length,".RData"))
```

The following R code was used for loess regression:

```
f <- read.table("CrossKingdom_LOWESS_wo_CRISPR.csv", header = TRUE, sep = ";")
df$Category <- factor(df$FeatureOrigin, c("Natural", "Synthetic"))
p<-ggplot(data=df, aes(x=PartLength, y=GeneticDistance, group=Category, shape=Category,
color=Category)) +
        theme(  panel.grid.major.x = element_blank(),
                      panel.grid.major.y = element_line(size=0.1, color="black"),
                      panel.background = element_rect(fill = "white"),
                      plot.background = element_rect(fill = "white", colour = "white"),
                      axis.text.x = element_text(color="black", size=8, angle=0,
family="Arial"),
                      axis.text.y = element_text(color="black", size=8, angle=0,
family="Arial"),
                      axis.title.y = element_text(margin = margin(t = 0, r = 10, b = 0, l = 0),
family="Arial"),
                      axis.title.x = element_text(margin = margin(t = 10, r = 0, b = 0, l = 0),
family="Arial"),
                      #axis.line = element_line(arrow = arrow(angle = 25, length = unit(0.2,
"cm"),ends = "last", type = "closed")),
                      axis.line = element_line(size=0.1, color="black"),
                      axis.ticks.y = element_blank(),
                      legend.position="top",
                      legend.text = element_text(color="black", size=8, angle=0,
family="Arial"),
                      legend.title = element_blank(),
                      legend.background = element_rect(fill = "white"),
                      legend.key = element_rect(fill = "white", colour = "white"),
                      plot.margin=unit(c(0,0.4,0.1,0.1),"cm")
              ) +

        scale_color_manual(values=c("#15a5d5", "#e84925")) +

        scale_fill_manual(values = c("#8edbf4","#f39f8d")) +

        scale_x_continuous(   name="Gene Length",
                                    breaks=seq(0,70000,1000),
                                    labels=seq(0,70000,1000),
                                    limits=c(0,70000),
```

```
                                    position="bottom") +

        scale_y_continuous(    name="Genetic Distance",
                                       breaks=seq(0,1,0.2),
                                       labels=seq(0,1,0.2),
                                       limits=c(0,10))  +

        stat_smooth(aes(fill=Category), method="loess", formula = y ~ x, level = 0.68, span = 
0.9) +
        coord_cartesian(xlim=c(0, 7000), ylim=c(0, 1))
```

The following Stata code was used for the regressions in Table 1:

```
OLS (1): regress GeneticDistance Synthetic if AntibioticResistance<1 & GeneticDistance>=0 & FusionProtein<1 & YearPlasmidReceived>2005 & YearPlasmidReceived<2016

OLS (2): regress GeneticDistance Synthetic PartLengthKB PartLengthKBXSynthetic if AntibioticResistance<1 & GeneticDistance>=0 & FusionProtein<1 & YearPlasmidReceived>2005 & YearPlasmidReceived<2016

OLS (3): regress CrossKingdom Synthetic PartLengthKB PartLengthKBXSynthetic if AntibioticResistance<1 & GeneticDistance>=0 & FusionProtein<1 & YearPlasmidReceived>2005 & YearPlasmidReceived<2016

Logit (4): regress CrossKingdom Synthetic PartLengthKB PartLengthKBXSynthetic if AntibioticResistance<1 & GeneticDistance>=0 & FusionProtein<1 & YearPlasmidReceived>2005 & YearPlasmidReceived<2016
```

The following Stata code was used for the regressions in Supplementary Table 12:

```
OLS (5): regress GeneticDistance Synthetic if AntibioticResistance<1 & GeneticDistance>=0 & FusionProtein<1 & YearPlasmidReceived>2005 & YearPlasmidReceived<2016 & CRISPR<1

OLS (6): regress GeneticDistance Synthetic PartLengthKB PartLengthKBXSynthetic if AntibioticResistance<1 & GeneticDistance>=0 & FusionProtein<1 & YearPlasmidReceived>2005 & YearPlasmidReceived<2016 & CRISPR<1

OLS (7): regress CrossKingdom Synthetic PartLengthKB PartLengthKBXSynthetic if AntibioticResistance<1 & GeneticDistance>=0 & FusionProtein<1 & YearPlasmidReceived>2005 & YearPlasmidReceived<2016 & CRISPR<1

Logit (8): regress CrossKingdom Synthetic PartLengthKB PartLengthKBXSynthetic if
AntibioticResistance<1 & GeneticDistance>=0 & FusionProtein<1 & YearPlasmidReceived>2005 &
YearPlasmidReceived<2016 & CRISPR<1
```

Supplementary Discussion

In this section, we provide a high-level workflow for how our sequence classification and phylogenetic distance estimation approaches can function within a more comprehensive approach to detect engineered organisms obtained from the environment. This operational context illustrates the complementary nature of our approaches with existing practices for biosurveillance. Further development of several of these steps, along with forensic attribution, is the subject of ongoing research efforts.

Workflow:

1. **Non-Native Organism Detection** (if an environmental sample is impure)
    a. 16srDNA sequence sample of interest to determine organism composition
    b. 16srDNA sequence relevant control sample(s) to determine baseline organism composition
    c. Compare compositions and any known environmental baselines to determine likelihood of non-native organism
2. **Synthetic and Transgene Detection** (after isolating organism of interest)
    a. **Next-Generation Sequencing**
    b. **De Novo Genome Assembly** or **Reference Genome Alignment**
    c. **ORF Detection**
    d. Transgene Detection:
        i. BLASTn sequences flanking ORF against RefSeq and record %QCov, %Id, and organism of top alignment
        ii. BLASTn ORF against RefSeq and record %QCov, %Id, and organism of top alignment
        iii. Compare organisms resulting from BLAST of ORF and from BLAST of flanking sequences.
            1. Are the organisms the same?
                a. If so, then gene is likely native to this organism
                b. If not, then **gene is likely non-native** and the **organism may be engineered**
            2. Are flanking sequences indicative of episomal expression?
                a. If so, then a potential transgene may be expressed episomally and origins of replication should be identified
                b. If not, then use genetic distance between organisms to gauge likelihood of natural horizontal gene transfer
    e. Synthetic Gene Detection:
        i. Is the top BLAST result for the ORF > 85 %Id and > 85 %QCov?
            1. If so, then gene is likely naturally occurring
            2. If not, then **gene is likely engineered** and **organism is likely engineered**
    f. Proceed to Next ORF

Supplementary References

1. Gilis, D., Massar, S., Cerf, N. J. & Rooman, M. Optimality of the genetic code with respect to protein stability and amino-acid frequencies. *Genome Biol.* **2,** research0049.1 (2001).

2. Galanie, S., Thodey, K., Trenchard, I. J., Filsinger Interrante, M. & Smolke, C. D. Complete biosynthesis of opioids in yeast. *Science* **349,** (2015).

3. Lanza, A. M. *et al.* A condition-specific codon optimization approach for improved heterologous gene expression in Saccharomyces cerevisiae. *BMC Syst. Biol.* **8,** 33 (2014).

4. Inouye, S., Sahara-Miura, Y., Sato, J. & Suzuki, T. Codon optimization of genes for efficient protein expression in mammalian cells by selection of only preferred human codons. *Protein Expr. Purif.* **109,** 47–54 (2015).
